# Simulation-Guided Analysis towards Trench Depth Optimization for Enhanced Flexibility in Stretch-Free, Shape-Induced Interconnects for Flexible Electronics

**DOI:** 10.3390/ma17153849

**Published:** 2024-08-03

**Authors:** Daniel Joch, Thomas Lang, Shawn Sanctis, Michael P. M. Jank

**Affiliations:** 1 Si Special Devices Group, Research and Development Semiconductor Devices, Fraunhofer Institute for Integrated Systems and Device Technology IISB, Schottkystrasse 10, 91058 Erlangen, Germany; 2 Electron Devices (LEB), Friedrich-Alexander-Universität Erlangen-Nuremberg, Cauerstr. 6, 91058 Erlangen, Germany

**Keywords:** stretchable electronics, mechanical design, structural design, numerical simulation, fabrication strategy, island-bridge

## Abstract

In this paper, we present an optimization of the planar manufacturing scheme for stretch-free, shape-induced metal interconnects to simplify fabrication with the aim of maximizing the flexibility in a structure regarding stress and strain. The formation of trenches between silicon islands is actively used in the lithographic process to create arc shape structures by spin coating resists into the trenches. The resulting resist form is used as a template for the metal lines, which are structured on top. Because this arc shape is beneficial for the flexibility of these bridges. The trench depth as a key parameter for the stress distribution is investigated by applying numerical simulations. The simulated results show that the increase in penetration depth of the metal bridge into the trench increases the tensile load which is converted into a shear force Q(x), that usually leads to increased strains the structure can generate. For the fabrication, the filling of the trenches with resists is optimized by varying the spin speed. Compared to theoretical resistance, the current–voltage measurements of the metal bridges show a similar behavior and almost every structural variation is capable of functioning as a flexible electrical interconnect in a complete island-bridge array.

## 1. Introduction

Building flexible electronics can be based on new material developments and advanced manufacturing technologies. As a result, soft electronic devices like soft transistors are made possible by using organic semiconductors and flexible dielectrics based on rubber or on solid-state polyelectrolytes. With this approach, device arrays can be built, and they are suitable for large area applications [1]. Nevertheless, island-bridge structures are a popular concept for building flexible electronics, where the islands, as the rigid part, are incorporated into a soft and stretchable material and connected by metal bridges to create the electrical functionality. Although it cannot compete with flexible material-based structures, where every component is stretchable, the island-bridge concept has the advantage that conventional high-performance devices can be placed on these islands, where they are isolated from strain [2,3].

There are different ways to build the flexible interconnects between the islands. One possibility is to fabricate the devices and metal lines in a standard process and transfer them onto a pre-stretched substrate. Releasing the pre-stretch pops up the bridges from surface, so that they have an arc-shaped structure and can tolerate a specific amount of strain [4,5,6]. By using trenches, which separate the islands from each other, the pre-stretch can also be used to let the metal lines buckle into the depth of the trench [7]. Of course, this transfer process is difficult to implement in a conventional processing route. Hence, there is also the possibility of using the silicon substrate itself and structure it from the front- and backside into islands [8]. The benefit of this approach is that the whole process stays within the standard planar technology, which simplifies handling and production. But on the other side, building 3D arc-shaped bridges using the trench depth is not an option anymore. Consequently, complex designs like mesh structures or combinations of specific beam structures must be used for the flexible interconnects, [8,9,10].

In this paper we show detailed investigations for our alternative manufacturing route which we use for fabricating stretch-free shape induced interconnects [11]. Here, the focus is on the trench depth as a key parameter. Numerical simulations were used to figure out the influence of the trench depth on the stress distribution of the metal bridge. An analytical model for explaining the stress behavior is established. Additionally, process optimization parameters for the fabrication of such advanced metal bridge structures are shown.

## 2. Materials and Methods

### 2.1. Integration Concept

**Figure 1:**
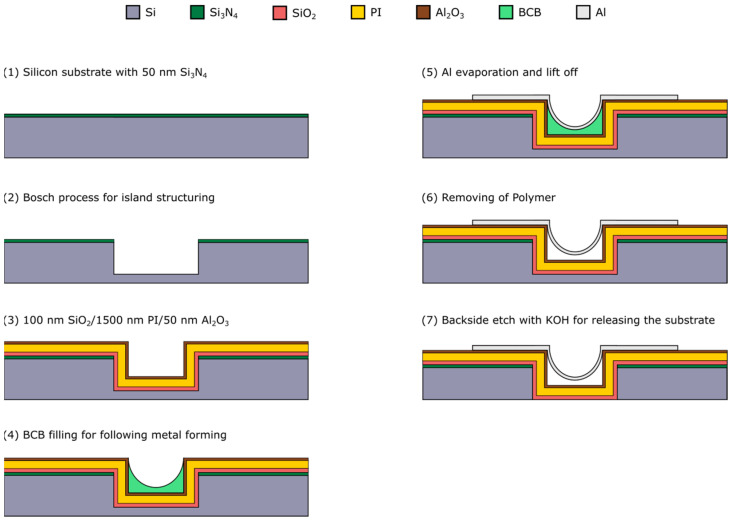
Complete process flow of the alternative manufacturing route [11].

In our previous paper we introduced the alternative concept of fabricating arc-shaped freestanding metal bridges into the trench depth (Figure 1). The 3D structures generated in this way serve as flexible interconnects between silicon islands and are fully integrated into the silicon planar technology. The concept also includes a flexible substrate (Figure 1, Polyimide) that simultaneously functions as a passivation layer [11].

Before structuring the trenches, it is also possible to fabricate electronic devices on the subsequently formed islands. After the Polyimide (PI) is spin coated, the metal lines are structured into the trench depth by using a forming process with a polymer, which is also spin coated. Finally, step 7 is used to remove the backside of the silicon wafer and to release the island-bridge substrate. With this last step the substrates also become semi-transparent.

The structures were investigated by using 2D simulations regard geometric parameters. While it turned out that the stress distribution is like that of bending under uniform line load, it also shows that the trench depth can have the most positive effect on stress distribution compared to metal width, thickness, and trench width. With the detailed investigations here, we want to give an explanation about this impact of the trench depth.

The forming process for the metal bridges was performed using Benzocyclobutene (BCB), which was spin coated into the trenches. The subsequent cleaning of the trenches to establish freestanding metal lines worked out, but also can cause damage to the interconnects, as the functionality test showed. All structures had a deviation in the resistance compared to a theoretical calculated value. Some interconnects did not work at all, which was a problem of defects. These results showed that an optimization of the process is needed. So, the focus of this work was set to step 4–6 of the manufacturing route (Figure 1).

### 2.2. Design of Numerical Modelling

**Figure 2:**
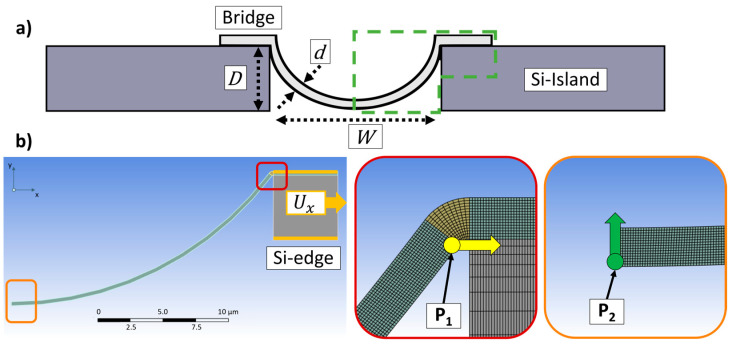
(**a**) Sketch of the concept to illustrate trench Depth *D*, trench width *W* and metal thickness *d*. The green box indicates the part for the simulation model; (**b**) example of a simulation model, here with trench depth of 10 µm (**left**). Illustration of *P_1_* and *P_2_* for the evaluation of the movement in *x* and *y* direction (**middle** and **right**).

The numerical simulations are performed using Ansys Mechanical 2022 R1 (Version 24.1.0.0) and with the same material parameters as in our first study [11]. Figure 2a shows a schematic drawing of the geometry illustrating the trench depth *D*, trench width *W* and the metal thickness *d*. While *W* and *d* were set constant at 40 µm and 300 nm, respectively, the trench depth *D* was varied to 5, 10, 15 and 20 µm. The simulations discussed here are focused on trench depth optimization, as it is anticipated to be the key factor for increasing the strain, which the metal can hold in the region of elastic deformation [11]. The upper limit of the elastic deformation is called the yield strength *γ_s_* and serves here as the maximum acceptable value for the stress in the bridge. The green box in Figure 2a displays the part of the concept, which was modeled in a symmetrical 2D simulation as can be seen in Figure 2b.

Since silicon is a rigid material, the entire island is not built in the model, but only the upper corner where the metal bridge arrives. As a load, a displacement *U_x_* is applied at this silicon edge to cause a stretching of the structure in *x* direction. First, the *U_x_* was set to 0.34 µm, which was sufficient to reach the yield strength *γ_s_* of the metal bridge over the 5 µm deep trench. In a second approach, depending on the trench depth *D*, a specific displacement to the silicon edge was applied to reach the *γ_s_* of the corresponding structure. The values for *U_x_* for the before mentioned trench depth variations were 0.34, 1.13, 2.57 and 3.98 µm. For both simulations, the movement in *x* direction of point *P_1_* at the upper end of the metal was compared to the movement in *y* direction for point *P_2_* in the middle (cf. Figure 2).

### 2.3. Material Processing

**Figure 3:**
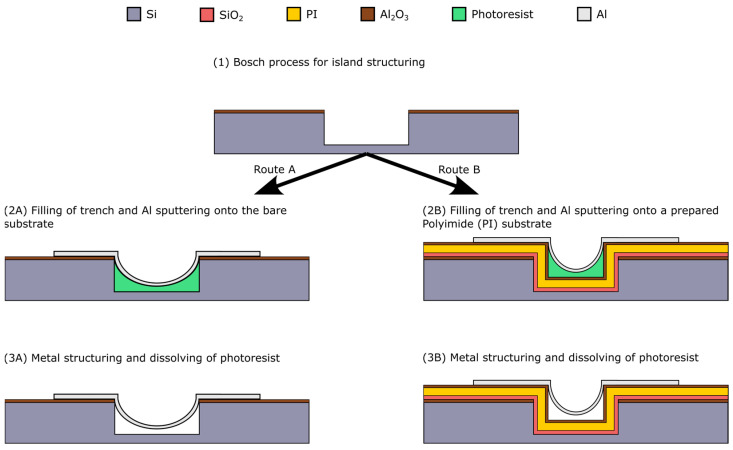
Material processing for the test structures: route A on bare silicon substrates, and route B on a Polyimide surface, which will be used in the reference process.

Here, as mentioned in Section 2.1, we focused our work on optimizing the metal deposition and the patterning. That means we reduced the complete process flow of Figure 1 to the fabrication scheme in Figure 3. So, we skipped the final step, which would be a wet chemical etching with potassium hydroxide (KOH) on the backside of the wafer, which is used to release the complete island–bridge substrate to make it flexible. Typically, array structures were fabricated in a way where the width of the trenches is kept constant at 40 µm, while the bridges vary between 5, 10, 20 and 30 µm in width. Figure 3 depicts the corresponding steps, wherein route A is focusing on the formation of the arc shape structure. For the filling of the trenches, a photoresist AZ5214 from MicroChemicals (Ulm, Germany) is used. To investigate the filling behavior, the resist was spin coated with 1000, 2000 and 3000 rpm and trench depths of 10 and 20 µm are used. Finally, the process is transferred to the reference process surface, which includes a PI layer to provide a flexible matrix for the islands (Figure 3, route B). To build these layers, a PI solution (PI 2611) of HD MicroSystems (Parlin, NJ, USA) is used, which is already solved with a thinner solution (T9039) by the supplier and is ready to use for spin coating processes. This PI is spin coated at 3000 rpm and then cured during a temperature ramp up of 300 °C, at which it is held for 1 h. Subsequently, 500 nm aluminum (Al) is deposited by sputtering and structured either by wet chemical or dry etching. For the wet chemical etching TechniEtch Al80 from MicroChemicals is used. The dry etching is performed in a Plasmalab System100 from Oxford Instruments (Abingdon, United Kingdom) with a mixture of chlorine (Cl) and hydrogen bromide (HBr). Thereafter, the resist is ashed in a TEPLA Plasma chamber from PVA TePla (Wettenberg, Germany) with an O_2_-Plasma at 300 W for 10 min, to yield/release freestanding metal bridges.

### 2.4. Electrical Characterization

The conductivity of the previously mentioned structures is verified by current-voltage measurements on interconnect loops over 96 islands. The structures were designed in a way so that one loop runs back and forth, placing contact 1 and contact 2 next to each other. The measurements are performed by applying a current sweep from −90 µA to +90 µA with steps of 20 µA, while measuring the required voltage between contact. The functionality of the structures is evaluated based on the determined resistance *R*, which is compared to theoretical resistance *R_theo_* of the bridges. The theoretical value is calculated by using the specific resistance of Al from the literature [12]. For the geometrical dimension of the structures, the length was taken from the mask layout, and for thickness, 500 nm was used. These current voltage measurements are an easy way to determine the yield of the bridges with regard to trench and metal width.

## 3. Results and Discussion

### 3.1. Numerical Simulations

**Figure 4:**
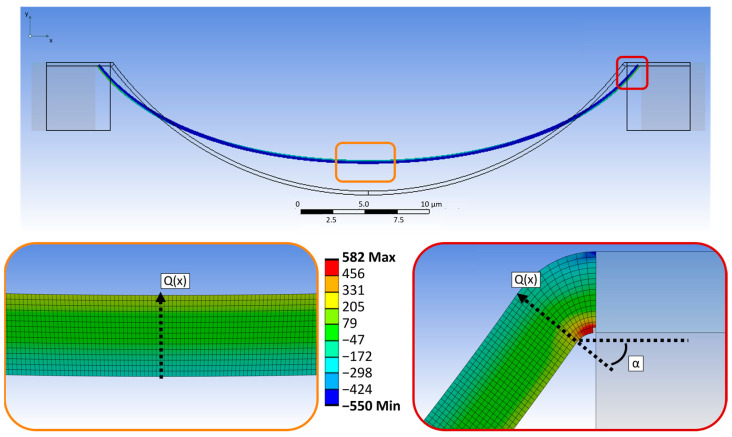
Behavior and maximum stress distribution of a metal bridge with 300 nm thickness and 5 µm width over a 10 µm deep trench with 40 µm width under tensile load.

Figure 4 shows the behavior of the bridge going through a trench 10 µm in depth under tensile load. The top image depicts a comparison of the deformed and undeformed model. As can be seen, the deformation in *x* direction causes a movement upwards of the middle section. In the detailed views the stress distribution along the metal is displayed. For the middle part (orange frame) the stress is positive (tensile) at the upper edge and negative (compressive) at the bottom. Contrary to this, at the support of the bridge (red), the tensile stress occurs at the bottom side and the compressive stress on the opposite. Such a stress distribution is also known from the pure bending of a straight beam under line load. It can be described as a uniaxial stress state, where the stress *σ_x_* at every position can be calculated with the following equation [13]:

(1) $$\sigma_{x}=\frac{M}{I_{y}}\cdot z=\frac{M}{{bh}^{3}}\cdot z$$

Here, *M* is the bending moment, *I_y_* the geometrical moment of inertia and *z* the distance to the neutral plane [13]. The yield strength *γ_s_* as the maximum tolerated value can be applied for *σ_x_* and the corresponding bending moment can be calculated. In our model *γ_s_* was set to 545 MPa which is a reported value for the targeted very thin metal lines (200–300 nm) [14]. Using Equations (2) and (3), together with the length *l* of the beam, the line load *q*(*x*) and the shear force *Q*(*x*) can be obtained:

(2) $$q(x)=\frac{-12M}{{(6x}^{2}-6x+l^{2})}$$

(3) $$Q(x)=\frac{-q}{2}(2x-l)$$

**Figure 5:**
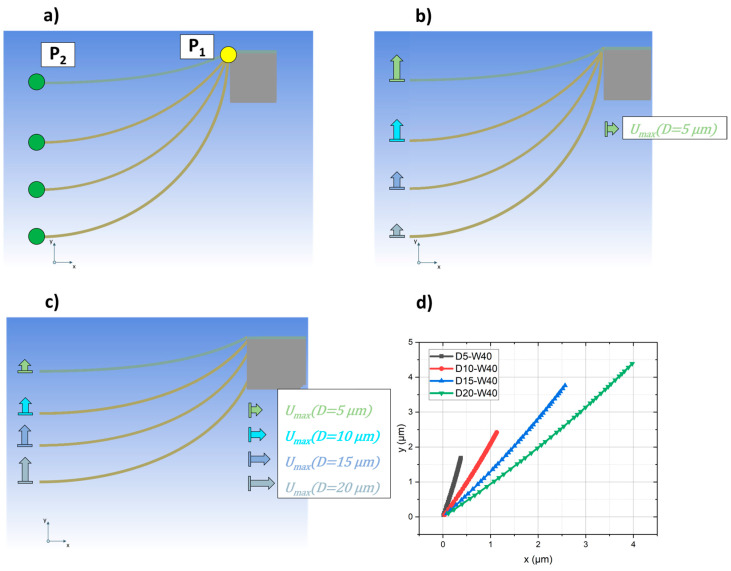
Results of the simulation to compare the movement of point P_1_ and P_2_ of the trench depth variation: (**a**) starting point without load; (**b**) deformation U_5µm_, which is sufficient to reach y_s_ of the bridge with a 5 µm deep trench; (**c**) individual load for every depth variation to reach y_s_; (**d**) plot of the y-position of P_2_ over the corresponding P_1_ displacement in x-direction for each bridge from (**c**).

For the derivation of these equations, a differential equation of the fourth order with specific boundary conditions is used to calculate a special bending case [13]. As can be seen in Figure 4, *γs* is the most likely to be reached on the upper end of the bridge at the transition to the island. Also, the direction of the shear force *Q*(*x*) and the angle between the horizontal plane and *Q*(*x*) is marked. Knowing these two components at exactly this point means that the force in x direction, which is caused by the applied movement, can be calculated via a trigonometric function. The details are given in Appendix A. Finally, the resulting deformation in *x* direction can be determined and used for the simulation to compare the movement of P1 and P2. The results are illustrated in Figure 5. For a better comparability in Figure 5a–c, the individual snapshots of each geometry variation were placed in one picture.

As described in Section 2.1 the simulation was carried out with bridges integrated into different trench depths *D*. So, the interconnect with *D* = 5 µm (D5-W40) is the flattest and therefore always the upper one, while the metal in the trench depth of 20 µm (D20-W40) is always the lowest. When the same displacement *U_x_* is applied to each model, as shown in Figure 5b, the movement in the *y* direction of the bridges is different, as illustrated by the arrows. It clearly decreases from sample D5-W40 to D20-W40. Figure 5c illustrates the case that a specific *U_x_* for each structure is applied. This time the *U_x_* is sufficient to reach the *γ_s_* of the corresponding interconnect. The movement in the *y* direction is also different, but this time it occurs opposite the case in Figure 5b. When the movement of P_2_ is plotted depending on P_1_, as shown in Figure 5d, it clearly can be seen that with increasing trench depth *D* the slope of the graph decreases. For sample D20-W40, it is almost equal to 1, at least until *U_x_* ≤ 3 µm. This movement of the middle part of the bridge is assumed to cause the bending of the metal and therefore also the specific stress distribution given in Figure 4. As described before, the tensile force F in *x* direction can be connected to the shear force *Q*(*x*) of the bending. Comparing the individual needed forces shows a decrease from trench depth 5 µm–20 µm. At the same time, when calculating the ratio *Q*(*x*)*/F*, it becomes apparent that with increasing trench depth, the ratio approximates to 1. For *D* = 20 µm, the tensile load is almost completely converted into the shear force *Q*(*x*). When the slope of the interconnect at the transfer to the island becomes larger, it is beneficial for the overall stress distribution and therefore for the achievable strain in the structure.

This also fits to the results, which we described in our first study about the design variations [11]. There, we also investigated if the stretchability of the interconnect can be improved by the trench width. In both cases, with the trench depth and the width, the initial length *L*_0_ of the metal line is increased. When calculating the strain with (*L* − *L*_0_)/*L*_0_, this should influence the reachable strain positively. But against this, we could only reach a strain of 5% with increasing the trench width, even though the *L*_0_ of the interconnect was comparable to the metal going through the trench 20 µm in depth [11]. This confirms our investigation in this study about the trench depth being a key parameter for the design of the metal because this improves the flexibility of the bridge the most.

### 3.2. Process Development and Implementation

**Figure 6:**
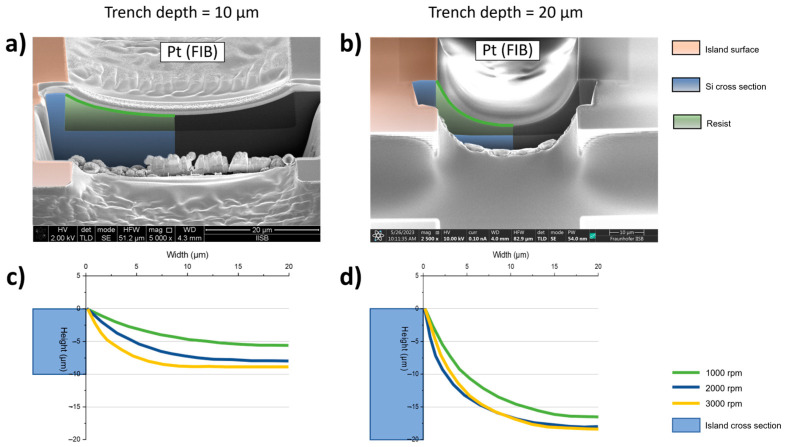
FIB cross sections of trenches with (**a**) 10 and (**b**) 20 µm depth with the green line indicating the arc shape profile of the resist spin coated with 1000 rpm for both; (**c**) extracted profiles of the resist in a trench with 10 µm depth, which was spin coated with 1000, 2000 and 3000 rpm into these trench depth variations; (**d**) extracted profiles in a 20 µm deep trench.

For the filling of the trenches with resist, the speed at which, during spin coating, the arc shape structure is achieved in the trenches is investigated. This is carried out for the 10 µm deep trenches as well as for the 20 µm depth. Figure 6a,b shows two examples of a cross section in the trench. The green line represents the profile of the resist, which is equal to the final form of the metal bridge. The profile for each spin speed is extracted for both trench depths. The diagrams in Figure 6c,d show the results. With increasing the spin speed, the resist becomes thinner in the middle of the trench, which means that the penetration depth for the metal bridge is increased. The largest difference in both trenches is between 1000 and 2000 rpm, while the depth for 2000 and 3000 rpm is almost the same. For a trench depth of 10 µm it can clearly be seen that with 3000 rpm the resist forms a plateau. In the 20 µm deep trench this also seems to start with 2000 and 3000 rpm. The aim for the final process is to establish a well-formed arc shape, so for producing, the parameters are set to 1000 and 2000 rpm for the 10 µm trench and to 1000 rpm for the 20 µm. Care must be taken about the wetting of the trench walls with resists. This becomes particularly significant at the trench depth of 20 µm. In the corresponding FIB image, the edge of the island is not covered. This is important for the layout of the bridges, meaning that the structures should be placed in the center of island and trench.

**Figure 7:**
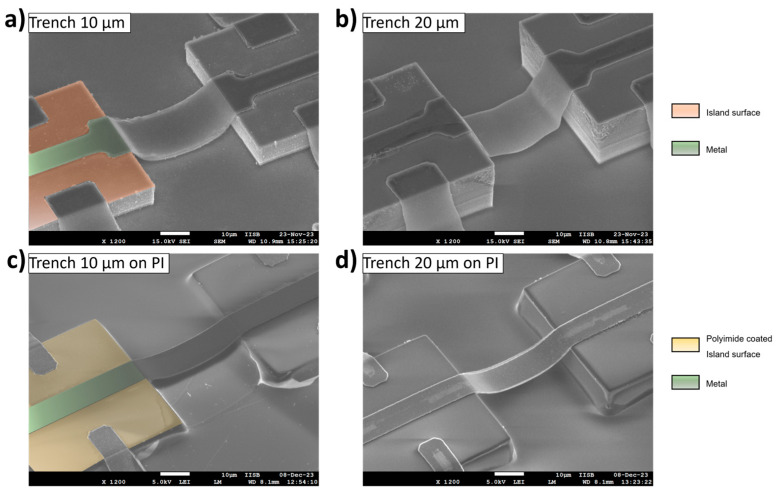
Freestanding metal bridges over trenches with 40 µm width: (**a**) Trench depth of 10 µm, (**b**) Trench depth of 20 µm, both without Polyimide coating and a metal width of 20 µm; (**c**) Trench depth of 10 µm; (**d**) Trench depth of 20 µm, both with Polyimide coating and a metal width of 10 µm.

In the next step, the freestanding metal bridges are formed on top of the resist coating. As described in Section 2.2, the structures are formed on a bare silicon surface and on Polyimide coated islands, which would be the final application. The results can be seen in Figure 7. The lithography and the structuring itself worked for a trench depth of 10 and 20 µm. On a bare silicon surface with *D* = 20 µm, the bridge is no longer as well formed as an arc shape, but the bridge penetrates quite well into the depth, which is also achieved for the 10 µm deep trench. Regarding the trench depth, it can be seen that it is significantly reduced with a PI coating. As with the simulations described, the trench depth is the key factor for generating more strain in the structures. Therefore, the PI filling must be taken into account as a design parameter for the trench depth, meaning that the trenches must be over-etched to achieve a specific trench depth in the final substrate.

### 3.3. Conductivity Measurements

**Figure 8:**
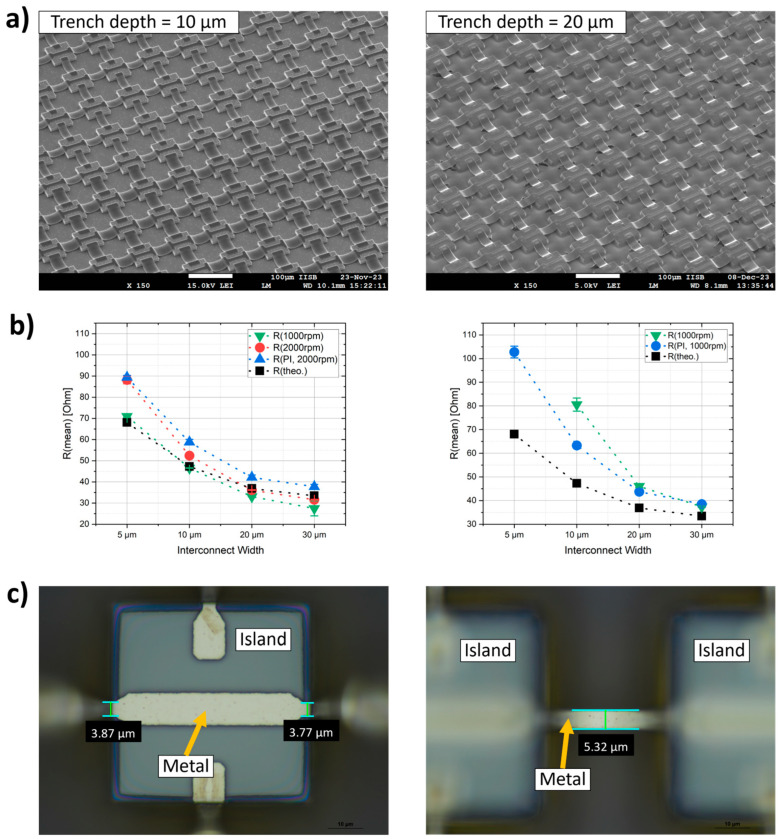
Examples for the island–bridge arrays with (**a**) 10 and 20 µm deep trenches and the metal width varying between 5, 10, 20 and 30 µm; (**b**) comparison of the determined resistance of each metal width and the theoretical values (10 µm trench depth on the right, 20 µm trench depth on the left), dotted lines are just for guidance; (**c**) problem of reduced metal width at the edge of the island as a consequence of the lithography.

To verify the functionality of the bridges, the fabricated island–bridge arrays are electrically characterized using I-V measurements. A mean value of resistance for each metal width is determined. The results are compared to the theoretical resistance. Examples for the arrays and the results of the functionality test can be seen in Figure 8a. The theoretical values show a decrease of the resistance with increasing the width of the metal, which can be expected because of the increased cross-sectional area. This can also be seen for all measured structures. For the trenches 10 µm in depth, the results are quite close to the theoretical values, especially the trench filling with 1000 rpm. There is an offset for the 5 µm width and 2000 rpm for both the bare silicon (green) surface and the PI coated (blue) one. For the 20 µm deep trenches, the results for the 5 and 10 µm widths are more broadly distributed. No value could be determined for the bare silicon surface with the 5 µm width, because no current flow could be detected. Figure 8c shows an example for one of these bridges over a 20 µm deep trench. It is assumed that scattering effects during the lithography, because of the island’s edge and the narrow structure width of the interconnect, cause this tapering of the final metal structure. As can be seen, the structure decreases from 5.3 µm to 3.8 and 3.9 µm, respectively. Additionally, as can be seen in Figure 7b, the slope of the arc shape metal structure near the island is very steep. This almost vertical profile can lead to a thinner metal during metallization, so that the thickness of 500 nm cannot be achieved. For these two reasons the cross-sectional area can be further decreased for these structures, which would cause an increase in resistance. Furthermore, it would make these structure very susceptible to defects, especially the bridges integrated to a trench depth of 20 µm. This is why no conductivity can be measured here (green curve in Figure 8b, right). For the PI coated islands this effect becomes less, and the structure width of 5 µm over a 20 µm deep trench is ok, but a larger difference to the theoretical resistance can be seen. As described, the PI decreases the trench depth and makes the edge of the silicon island smoother. Both can help in avoiding defects in the metal bridges. Consequently, for further optimization in the future, the layout of the narrow structures can also be adapted. For this purpose, the part that tapers on the island surface could be designed wider, so that this effect is already counteracted by a wider opening during lithography. The structure width in the trench can still be kept at 5 µm.

### 3.4. Comparative Analysis

The here-introduced manufacturing route of flexible interconnects can be categorized into structuring strategies for realizing stretchability. This category is often used in combination with rigid devices [15]. Within these comparable, other strategies are wavy structures, which can be divided into coplanar (serpentines) and noncoplanar (buckled or arc-shape and helical), and origami or kirigami structures [16]. Serpentines are typical 2D structures, and their stretchability depends on the width, the wavelength, and the amplitude. By optimizing this they can reach strains up to 70% with out-of-plane twisting [16]. They can be fabricated by standard metal deposition and photolithography [15]. Also, the already mentioned more-complex interconnects like mesh structures or specific beam designs can be seen as a further development in this field. They can be stretched 100% or bent 360° [8,9]. The buckling strategy, which includes the helical interconnects, is also mentioned in the introduction, and builds the basic strategy for 3D concepts. Simple structures start with a strain tolerance of 17%, but this can easily be improved by periodic buckling of up to 150% [6,15]. The basic process for building these 3D structures is using a pre-stretched elastic substrate, on which the previously fabricated structures can be transferred. Releasing the pre-stretch forms the out of plane metal lines. Cutting and folding is used with the kirigami and origami strategies. With kirigami, patterned cutting lines are used to delocalize the stress, which leads to strains of 290% by out-of-plane deflection. This can be further increased to 400% by using materials based on nanowires. Origami means to fold a structure in a periodic pattern, which also distributes the applied load. Depositing a metal film on such a structure can lead to a strain tolerance of 30%. Analyzing the optimized structure (a bridge with 5 µm width through a 20 µm deep trench) of our introduced interconnects, the stretchability is around 25% [11]. This means it is much lower than most of the other structures. But, the great advantage is the complete integration into silicon planar technology, which only the serpentines can compete with [15]. This eliminates special process steps and lowers process complexity. The pre-stretch processes, cutting (kirigami) or molding structures (origami) cannot offer this. Also, using alternative material composites like nanowires or conducting polymers cannot be handled in a standard manufacturing route and, additionally, they show decreased electrical performance [2]. With the standard Si technology combined with the island bridge concept, the usual high-performance materials are still available. Furthermore, our concept can also be understood as a technology platform that can be extended for additional structuring. By integrating serpentines and mesh interconnects into the trench depth, these structures can be transferred from 2D to 3D, which would offer an additional method for strain compensation.

Using the standard technology of a cleanroom production line also offers the advantage of scalability. By implementing the Polyimide at a very late stage of the process, everything before that can also see high temperature steps. This is comparable to the so called “polymer-last process” of Mimoun et al., which they use to build 2D flexible sensor devices based on silicon wafers [9]. Some restrictions can be made because of the final chip size, which is needed for possible future applications, and for the wafer dimensions. For the backside etching step, which is needed to release the final structure, it is easier to use thinned wafers (thickness of 300 µm or less). Handling these wafers is more difficult and the risk of damaging them is greater, especially with increasing wafer diameters. This means that smaller wafers are practicable, and therefore, the chip size required for a possible future application should be moderate, such that a corresponding number can be processed in parallel.

## 4. Conclusions

Based on numerical modelling, we have successfully demonstrated an optimized manufacturing route for flexible interconnects, which uses the trench depth as a design parameter which can be completely integrated into the standard silicon planar technology. Detailed investigations on the trench depth simulations clearly showed that by increasing the slope of the metal bridge when reaching the island, more tensile load is converted into shear force Q(x). This effect improves the overall stress distribution during tensile load.

To implement this structure in the manufacturing process, the filling of the trenches with resists works well over a complete island-bridge array with a size of 0.5 × 0.5 cm. While both trench depths can be filled with 1000 rpm during spin coating, the filling with 2000 rpm leads to even better arc shape structures in the 10 µm deep trench.

Current–voltage measurements show a decrease of resistance with increasing width, which fits the estimated theoretical resistance values. Although some deviations in the practical measured values to the theoretical ones can be expected, it must be emphasized that all metal interconnects on the final PI coated wafers are found to be completely operational, demonstrating the scalability of this approach.

A brief comparative analysis shows that the stretchability tends to be in the lower range, but that our presented process has advantages in terms of processability. The standard silicon planar technology offers access to high performance materials and batch processing, which helps to scale up the production of such structures.

## Figures and Tables

**Figure A1:**
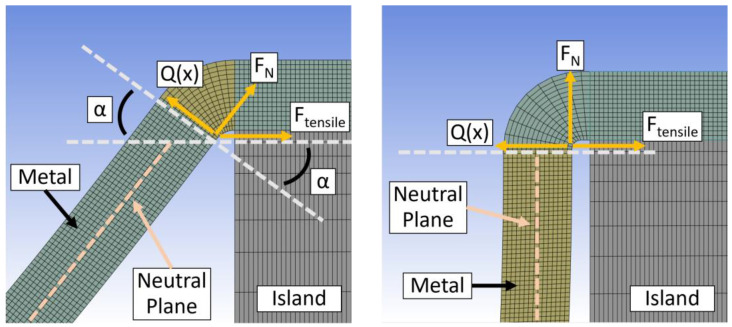
Description of correlation of forces at the critical point at the end of the metal bridge: example for a bridge through a 40 µm long a 10 µm deep trench (**left**), trench with width = 40 µm and height = 20 µm (**right**).

## Data Availability

The original contributions presented in the study are included in the article, further inquiries can be directed to the corresponding author.

## Appendix A

Here, the derivation for the equations from Section 3.1 are shown. The following calculations are based on [13,17].

With the assumption of a uniform surface load, there is the general differential equation of the fourth order [13,17]:

(A1) $${EIw}^{\mathrm{IV}}=q\left(x\right)$$

Here, *E* is the young’s modulus, *I* the geometrical moment of inertia, *w* the curvature and *q* the surface load. The *x* coordinate describes a specific point along the beam and is in the range of 0 ≤ *x* ≤ l, where *l* is the maximum length of the beam. Integrating Equation (A1) leads to the four constants:

(A2) $$\mathrm{EIw}\left(x\right)=q\frac{x^{4}}{24}+c_{1}\frac{x^{3}}{6}+c_{2}\frac{x^{2}}{2}+c_{3}x+c_{4}$$

The constants can be solved with the boundary condition that the beam is clamped at both ends [13]:

$$w\left(0\right)=0$$

$$w’\left(0\right)=0$$

$$w\left(l\right)=0$$

$$w’\left(l\right)=0$$

(A3) $$\mathrm{EIw}\left(x\right)=q\frac{x^{4}}{24}+ql\frac{x^{3}}{6}+ql^{2}\frac{x^{2}}{2}$$

With the second derivation of Equation (A3), the bending moment *M*(*x*) can be calculated [13,17]:

(A4) $$\mathrm{EIw}’x=-M\left(x\right)=\frac{q}{12}(6x^{2}-6lx+l^{2})$$

And with the derivation of *M*(*x*), the shear force *Q*(*x*) can be calculated:

(A5) $$Q\left(x\right)=\frac{q}{2}(2x-l)$$

As described in our results, the most critical point for the stress in the metal is the point next to the edge of the island. And, we mentioned that the stress should not overcome the yield strength *γ_s_*.

When using *γ_s_* as *σ_x_* in Equation (1) from Section 3.1, there is a second possibility to calculate *M*(*x*). After that, *M*(*x*) and Equation (A4) can be used to find *Q*(*X*), which is needed in Equation (A5) for *Q*(*X*).

When *Q*(*X*) is solved in this way, the correlation of forces can be drafted as can be seen in Figure A1.

There is the shear force *Q*(*x*) and the normal force F_N_, which come from the bending of the structure [13]. The third one is the tensile force F_tensile_, because of the deformation in x direction. Finally, to find F_tensile_, the standard trigonometric functions can be used. Therefore, the angle α must be determined from the model by measuring it during the building of the model. As illustrated in Figure A1, the angle α decreases with the increasing of the slope of the metal bridge. For the 20 µm deep trench (Figure A1, right), it can be assumed as 0. So, F_tensile_ can be calculated for every geometric variation and be used for simulations.

## References

[1] Liu Z., Zhao Y., Yin Z. (2024). Low-power soft transistors triggering revolutionary electronics. Innovation.

[2] Yin L., Lv J., Wang J. (2020). Structural Innovations in Printed, Flexible, and Stretchable Electronics. Adv. Mater. Technol..

[3] Matsuhisa N., Chen X., Bao Z., Someya T. (2019). Materials and structural designs of stretchable conductors. Chem. Soc. Rev..

[4] Xue Z., Song H., Rogers J.A., Zhang Y., Huang Y. (2020). Mechanically-Guided Structural Designs in Stretchable Inorganic Electronics. Adv. Mater..

[5] Li H., Ma Y., Huang Y. (2021). Material innovation and mechanics design for substrates and encapsulation of flexible electronics: A review. Mater. Horiz..

[6] Kim D.H., Song J., Choi W.M., Kim H.S., Kim R.H., Liu Z., Huang Y.Y., Hwang K.C., Zhang Y.W., Rogers J.A. (2008). Materials and noncoplanar mesh designs for integrated circuits with linear elastic responses to extreme mechanical deformations. Proc. Natl. Acad. Sci. USA.

[7] Lee J., Wu J., Ryu J.H., Liu Z., Meitl M., Zhang Y.W., Huang Y., Rogers J.A. (2012). Stretchable Semiconductor Technologies with High Areal Coverages and Strain-Limiting Behavior: Demonstration in High-Efficiency Dual-Junction GaInP/GaAs Photovoltaics. Small.

[8] Sosin S., Zoumpoulidis T., Bartek M., Wang L., Jansen K.M.B., Ernst L.J. Mesh Interconnects for Silicone Embedded Stretchable Silicon Electronics. Proceedings of the 10th Electronics Packaging Technology Conference (EPTC).

[9] Mimoun B., Henneken V., van der Horst A., Dekker R. (2013). Flex-to-Rigid (F2R): A Generic Platform for the Fabrication and Assembly of Flexible Sensors for Minimally Invasive Instruments. IEEE Sens. J..

[10] Shafqat S., Hoefnagels J.P., Savov A., Joshi S., Dekker R., Geers M.G. (2017). Ultra-Stretchable Interconnects for High-Density Stretchable Electronics. Micromachines.

[11] Joch D., Lang T., Sanctis S., Jank M.P. Stretch-Free, Shape-Induced Interconnects for Flexible Electronics Via an Island-Bridge Fabrication Process. Proceedings of the 20th International Conference on Experimental Mechanics.

[12] Rumble J. (2022). Electrical Resistivity of Pure Elemental Metals as a Function of Temperature. CRC Handbook of Chemistry and Physics.

[13] Richard H.A., Sander M. (2008). Biegung von Balken und balkenartigen Tragwerken. Technische Mechanik. Festigkeitslehre.

[14] Soare S., Bull S.J., Oila A., O’Neill A.G., Wright N., Horsfall A., dos Santos J. (2003). Determination of mechanical parameters for rotating MEMS structures as a function of deposition method. MRS Online Proc. Libr..

[15] Lee B., Cho H., Jeong S., Yoon J., Jang D., Lee D.K., Kim D., Chung S., Hong Y. (2022). Stretchable hybrid electronics: Combining rigid electronic devices with stretchable interconnects into high-performance on-skin electronics. J. Inf. Disp..

[16] Hu H., Zhang C., Ding Y., Chen F., Huang Q., Zheng Z. (2023). A Review of Structure Engineering of Strain-Tolerant Architectures for Stretchable Electronics. Small Methods.

[17] Balke H. (2010). Reine Biegung gerader Balken. Einführung in Die Technische Mechanik.

